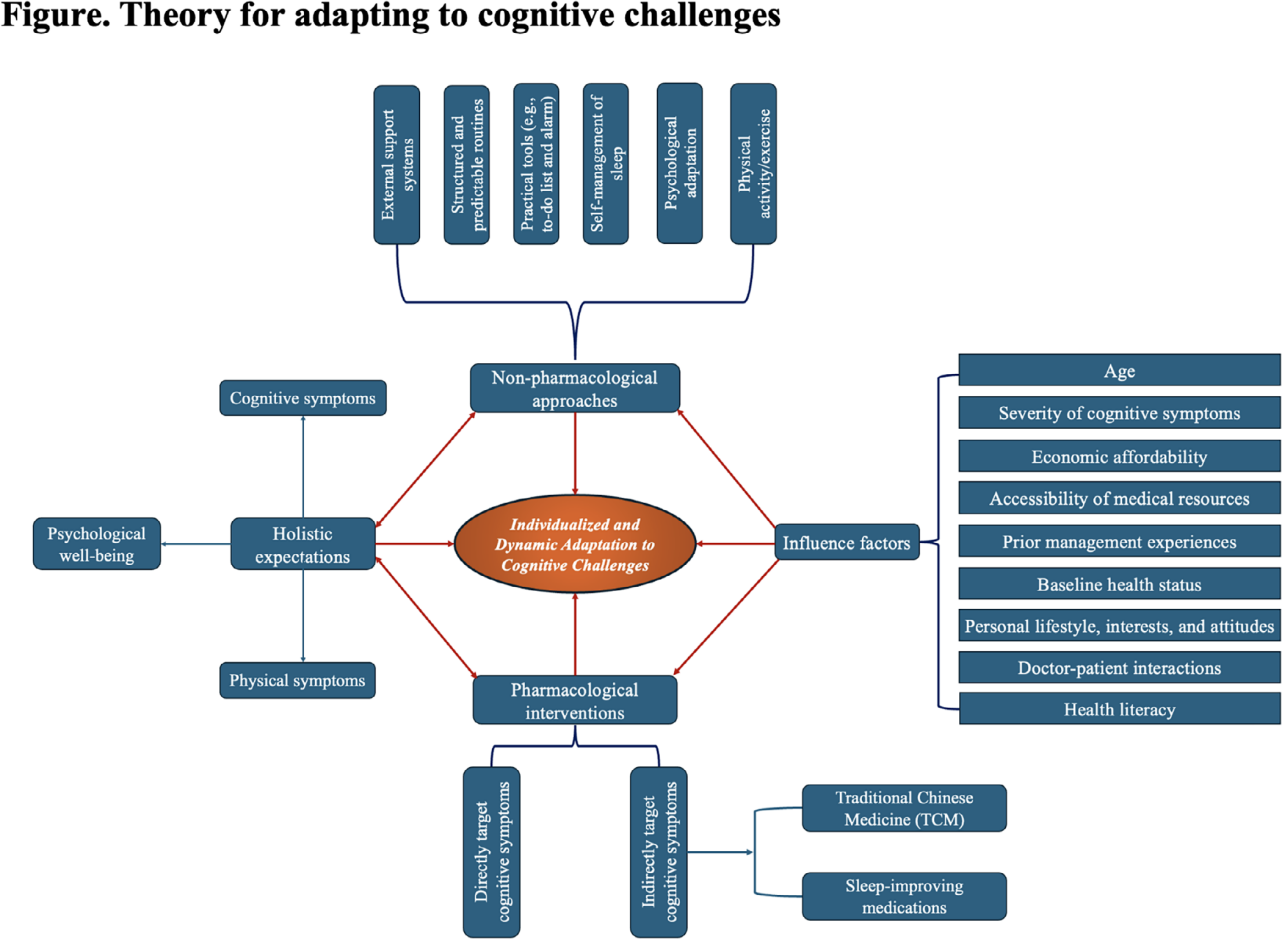# Treatment Experiences, Preferences, and Expectations for Long COVID Cognitive Impairments: A Constructivist Grounded Theory Study

**DOI:** 10.1002/alz70858_101300

**Published:** 2025-12-25

**Authors:** Dan Shan, Carol Holland, Trevor Crawford

**Affiliations:** ^1^ Lancaster University, Lancaster, Lancaster, United Kingdom

## Abstract

**Background:**

Long COVID‐associated cognitive impairments significantly diminish patients' quality of life. However, there is a lack of research exploring patient perspectives on treatment preferences and expectations, especially across age groups.

**Method:**

In this qualitative study, we employed a constructivist grounded theory approach. Semi‐structured online interviews were conducted with 23 Chinese participants, comprising 10 young adults (aged 18‐39 years) and 13 older adults (aged ≥60 years).

**Result:**

A central theoretical framework of *‘Individualized and Dynamic Adaptation to Cognitive Challenges’* was developed, illustrating how factors including age, severity of cognitive symptoms, economic affordability, accessibility of medical resources, prior management experiences regarding cognitive symptoms, baseline health status, personal lifestyle, interests, and attitudes, doctor‐patient interactions, and health literacy shaped participants’ preferences and expectations. ‘Preference for non‐pharmacological interventions’ emerged as the core theme for young adults, consisting of subthemes including self‐directed strategies (formed by concepts such as psychological adaptation and practical tools like to‐do lists and alarms) and emotional and psychological support. In contrast, ‘balanced use of pharmacological interventions’ was identified as the core theme for older adults, consisting of non‐pharmacological interventions (formed by concepts such as family support and structured and predictable routines), pharmacological interventions, and holistic expectations (formed by the concept that address not only cognitive but also physical and emotional well‐being). Across both age groups, improving sleep quality and psychological well‐being was consistently highlighted as essential for effective management. Disrupted sleep was viewed not only as a symptom but also as a factor that could exacerbate cognitive symptoms. For older adults, the need for psychological support often stemmed from poorer baseline health and feelings of neglect and isolation due to limited family presence in daily life. By comparison, young adults expressed a need for emotional care primarily driven by the stigma surrounding long COVID, particularly the perceived lack of trust and understanding from older generations and broader society.

**Conclusion:**

Our study is the first to qualitatively explore and compare age‐specific treatment preferences, experiences, and expectations regarding long COVID‐associated cognitive impairments. By highlighting the heterogeneity of age‐specific preferences and expectations, it provides a comprehensive theoretical framework to inform the development of dynamic, patient‐centred interventions.